# Ultra-short term HRV features as surrogates of short term HRV: a case study on mental stress detection in real life

**DOI:** 10.1186/s12911-019-0742-y

**Published:** 2019-01-17

**Authors:** R. Castaldo, L. Montesinos, P. Melillo, C. James, L. Pecchia

**Affiliations:** 1 School of Engineering, University of Warwick, CV47AL, Coventry, UK; 2 Institute of Advanced Studies, University of Warwick, CV47AL, Coventry, UK; 3 Multidisciplinary Department of Medical, Surgical and Dental Sciences, University of Campania Luigi Vanvitelli, Naples, Italy

**Keywords:** Heart rate variability (HRV), Ultra-short term HRV analysis, Mental stress detection, Data-driven machine learning

## Abstract

**Background:**

This paper suggests a method to assess the extent to which ultra-short Heart Rate Variability (HRV) features (less than 5 min) can be considered as valid surrogates of short HRV features (nominally 5 min). Short term HRV analysis has been widely investigated for mental stress assessment, whereas the validity of ultra-short HRV features remains unclear. Therefore, this study proposes a method to explore the extent to which HRV excerpts can be shortened without losing their ability to automatically detect mental stress.

**Methods:**

ECGs were acquired from 42 healthy subjects during a university examination and resting condition. 23 features were extracted from HRV excerpts of different lengths (i.e., 30 s, 1 min, 2 min, 3 min, and 5 min). Significant differences between rest and stress phases were investigated using non-parametric statistical tests at different time-scales. Features extracted from each ultra-short length were compared with the standard short HRV features, assumed as the benchmark, via Spearman’s rank correlation analysis and Bland-Altman plots during rest and stress phases. Using data-driven machine learning approaches, a model aiming to detect mental stress was trained, validated and tested using short HRV features, and assessed on the ultra-short HRV features.

**Results:**

Six out of 23 ultra-short HRV features (MeanNN, StdNN, MeanHR, StdHR, HF, and SD2) displayed consistency across all of the excerpt lengths (i.e., from 5 to 1 min) and 3 out of those 6 ultra-short HRV features (MeanNN, StdHR, and HF) achieved good performance (accuracy above 88%) when employed in a well-dimensioned automatic classifier.

**Conclusion:**

This study concluded that 6 ultra-short HRV features are valid surrogates of short HRV features for mental stress investigation.

**Electronic supplementary material:**

The online version of this article (10.1186/s12911-019-0742-y) contains supplementary material, which is available to authorized users.

## Background

Stress is defined by the American Psychological Association as “the pattern of specific and nonspecific responses an organism makes to stimulus events that disturb its equilibrium and tax or exceed its ability to cope” [1]. In particular, mental stress has been defined by Lazarus and Folkman as a form of stress that occurs because of how events in one’s external or internal environment are perceived, resulting in the psychological experience of distress and anxiety [2, 3]. In humans, mental stress has been investigated using several cognitive stressors in laboratory (e.g., computer work tasks, Stroop color word test, arithmetic tasks, game tasks) or in real-life scenarios (e.g., public speech tasks, academic examinations, during surgeries) [4]. Mental stress can manifest itself as many different symptoms and signs, ranging from physiological (i.e., increased heart rate, sweating) to psychological (i.e., anxiety) and behavioral (i.e., altered sleep patterns) manifestations. Moreover, subjects may experience these to varying degrees [5, 6]. In this study, mental stress is investigated during a verbal academic examination, which has shown to be a stressful situation resulting in accentuated sympathovagal antagonism [7–9]. Although different concerns have been raised about academic examination as stressor due to arousal or other feelings, it has shown to be a reliable cognitive stressor [8–10]. In this study, the rest phase was acquired in a period where subjects were not under any academic pressure.

Mental stress has been investigated in various fields due to its detrimental effects on the daily routine [11]. In fact, whereas some kinds of stress may be beneficial by allowing humans to respond to threats in their environment, mental stress can also decrease attentional resources, impair working memory and memory retrieval, and overload cognitive systems [6, 12]. Stress influences judgment and decision-making, and has been shown to reduce human performance [4, 13]. There is a need to better understand the impact of stress on cognition and performance, especially in high-risk domains such as military, policing, surgery, aviation, driving, and elite-level sport, in which risks or threats are prevalent and they can result in devastating consequences [4, 14].

Although in the existing literature there are multiple physiological signals used to detect stress, such as galvanic skin response, blood pressure, electroencephalogram, respiration rate, and electrocardiogram (ECG), heart rate variability (HRV) is currently one of the most investigated methods for assessing mental stress [4]. Moreover, HRV is a more sensitive measure of stress than heart rate alone [15]. HRV describes the variations of the intervals between consecutive peaks of the R-waves in an ECG and it can be analyzed in the time, frequency and non-linear domains. HRV analysis can be performed on 24 h nominal recordings (defined as long term HRV analysis), 5 min recordings (defined as short term HRV analysis) or shorter recordings [16]. In this paper, ultra-short term HRV analysis is defined as the analysis performed on HRV excerpts shorter than 5 min.

During mental stress, there is an activation of the sympathetic nervous system and a withdrawal of the parasympathetic nervous system [5], which results in significant changes in many HRV features [4, 17]. Previous studies have shown that long and short HRV features change consistently during mental stress and that they are able to reliably capture stress in laboratory and real-life scenarios [4, 5, 18–20]. However, much less work has been done on real-life stress detection via ultra-short term HRV analysis. The demands of ultra-short term HRV analysis for monitoring individual’s well-being status is increasing, due to the diffusion of wearable sensors in the healthcare and consumer devices such as mobile phones and smart watches [21, 22]. In e-health monitoring, in fact, the conventional 5 min recordings might be unsuitable, due to real-time requirements. Ultra-short term HRV analysis, especially in combination with wearable sensors, may allow continuous and real time monitoring of an individual’ stress levels, which is important in some circumstances or jobs (e.g., surgeons, airplane pilots). However, numerous challenges have arisen by shortening HRV excerpts below 5 min. In fact, a recent literature review highlights the lack of rigorous methods utilized to explore the extent of which ultra-short HRV features can be used to estimate short term ones [22]. In medicine, particularly in clinical trial designs, in order to cope with this kind of problems, the concept of surrogate endpoint (or marker) was introduced [23, 24]. However, proving whether a marker is a valid surrogate of a real clinical outcome can be quite difficult, and combination of appropriate statistical and correlation tests is required, as detailed elsewhere [22]. In a previous study [25] we explored the feasibility of using ultra-short HRV features for mental stress automatic detection basing on descriptive statistics and without developing a systematic method to identify reliable surrogates for short HRV features.

To the best of the authors’ knowledge, none of the studies investigating ultra-short HRV features has proposed a robust methodology to assess if ultra-short HRV features are valid surrogates of short ones to detect stress [22]. There have been some attempts to investigate the reliability and accuracy of ultra-short term HRV analysis [15, 23, 24, 26–42], but only one study investigated the validity of ultra-short HRV features in a more rigorous way [39]. However, the authors in [39] only considered 2 time domain HRV features under one standard condition (i.e., rest phase).

Therefore, the current study is the first proposing a rigorous method to assess the validity of ultra-short HRV features for detecting mental stress. The current paper aims to show to what extent HRV features are reliable and accurate to automatically detect mental stress when moving from short (used as benchmark) to ultra-short term HRV analysis. Moreover, the proposed method could be suitable for other applications using ultra-short term HRV analysis to detect an adverse healthcare event.

## Methods

### Dataset

The data analyzed in the current study were acquired from 42 healthy students in the School of Biomedical Engineering of the University of Naples Federico II (Italy) as described in a previous study [7]. Melillo et al. assumed that verbal university examinations was a valid real-life stressor [7]. This hypothesis was previously proposed by other studies [8, 9] and confirmed by a systematic literature review proving that HRV features, computed during a verbal examination, presented a similar behavior as those acquired using other stressors [4]. As described in Melillo et al. [7], the data were acquired in two different days: the first recording was performed during an ongoing university verbal examination (i.e., stress phase) before Easter break (which in Italy last less than 10 days), while the second one was taken in controlled resting condition (i.e., rest phase) after the vacations, far away from stress induced from study routines. During the rest phase, subjects were invited to sit on a comfortable chair and they were induced to talk, as they had done during the verbal examination, as talk has proven to alter respiration and therefore, HRV features [43]. Ethical permission was sought from the Local Ethic Committee. A commercial electrocardiograph (Easy ECG Pocket, manufactured by Ates Medical) was used to acquire 3-lead clinical research ECG signals, with a sampling frequency of 500 Hz and a resolution of 12 bits per sample. Kendall™ 530 series foam electrodes were used as they are designed for superior performance including adult stress, holter and diaphoretic applications. Electrodes were placed to the subjects’ chest according to the standard guidelines [44]: one electrode was placed under right clavicle near right shoulder within the rib cage frame; the second electrode was placed under left clavicle near left shoulder within the rib cage frame; the third electrode was placed on the left side below pectoral muscles lower edge of left rib cage. Subjects were helped by expert staff of their gender to position the electrodes.

The resting condition was measured at the same time slot as for the stress phase, in order to minimize circadian cycle effects on the HRV, and in the same menstrual cycle for women, as this is also a relevant measure for HRV features [7, 45]. The participants were examined under standard conditions during rest and stress phases: in the same quiet room, at a comfortable temperature, while sitting. Data acquisitions were carried out in the morning for both rest and stress phases between 9 a.m. and 12 p.m.

Three-lead ECG was recorded for at least 30 min. The first 15 min (i.e., adaptation time) were excluded and one ECG excerpt of 5 min was extracted and analyzed.

Participants were invited to refrain from drinking alcohol and to limit their tea and coffee intake (2 cups max.) in the 24 h prior to the acquisitions, as alcohol, caffeine and tea have shown to alter HRV features [46].

The participants enrolled in the study had no history of heart disease, systemic hypertension, metabolic disorders or other diseases potentially influencing HRV. They were not obese and did not consume medication, drugs or alcohol in the 24 h preceding the experiments. All of the participants signed specific informed consent form before the acquisitions. More details on the protocol can be found in [7].

### HRV analysis

**Fig. 1:**
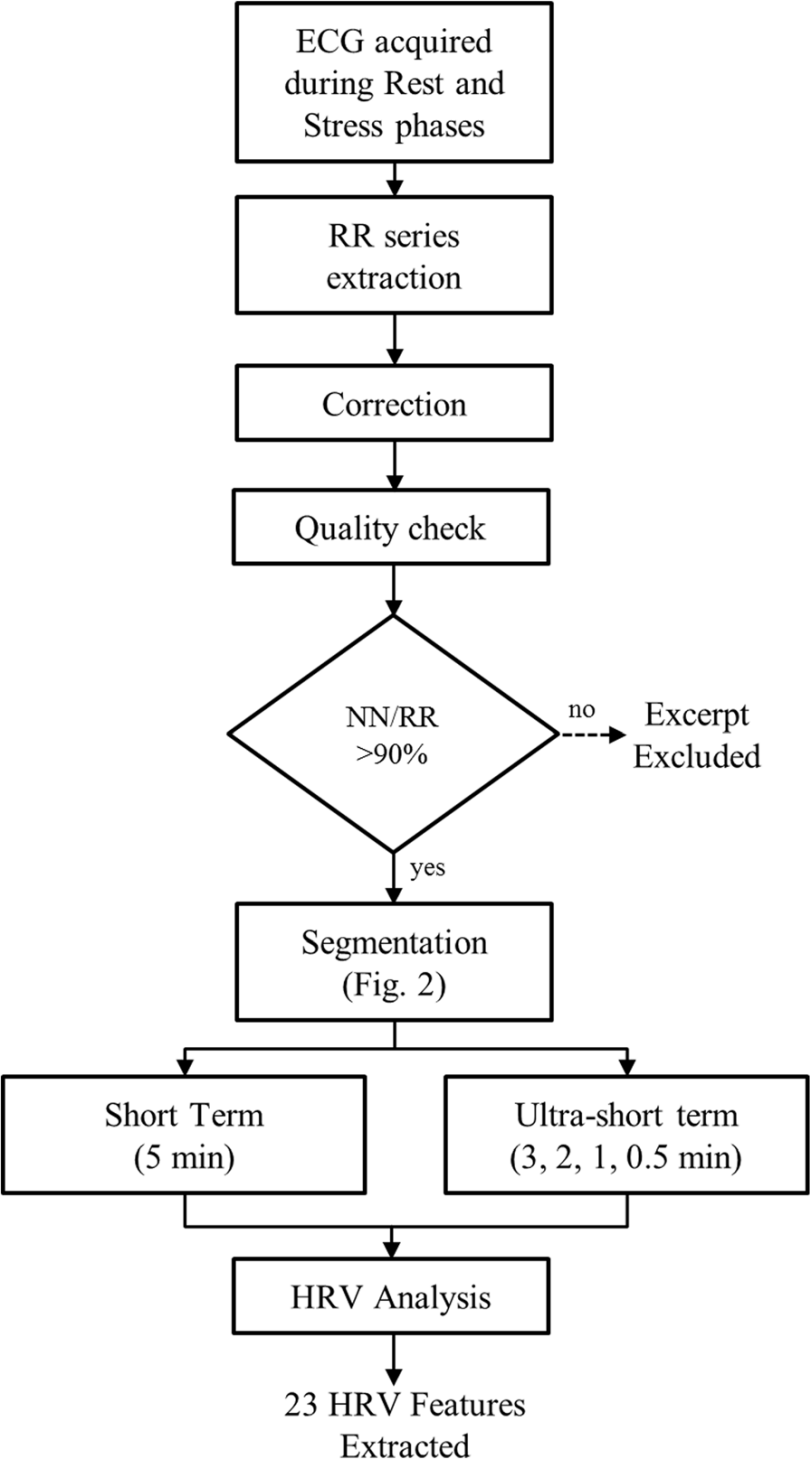
HRV processing workflow. ECG: Electrocardiogram; NN/RR is the ratio of the total RR intervals labelled as NN (normal-to-normal beats); short term: HRV is analyzed in 5 min excerpts; ultra-short term: HRV is analyzed in excerpts of 3, 2, 1 and 0.5 min length

As shown in Fig. 1, the RR interval time series were extracted from ECG recordings using an automatic QRS detector, WQRS, available in the PhysioNet’s toolkit [47], based on nonlinearly scaled ECG curve length features. Details on ECGs pre-processing used in the PhysioNet’s toolkit can be read in Zong et al. [48]. The automatic QRS detection was followed by visual inspection and manual correction. QRS review and correction was performed using WAVE, which is the graphical user interface to visualize biomedical signals provided by PhysioNet and includes facilities for interactive annotation editing. Additional details can be found elsewhere [49].

The fraction of total RR intervals labelled as normal-to-normal (NN) intervals was computed as NN/RR ratio. When ectopic beat correction methods are not adopted, and more than one RR excerpt is available per each subject, the NN/RR ratio is used to identify a window of time of sufficient quality, excluding those windows of time in which this ratio is lower than a threshold. Thresholds of 80% [47] and 90% [50] have been proposed. In studies enrolling only healthy and young subjects, a lower NN/RR ratio is associated with movement artifacts. In the current study, in which subjects were healthy and young, sitting in a comfortable position, a threshold of 90% was chosen and still no records were excluded. Therefore, short HRV features were computed from the first 5 min after the adaptation time for all of the participants.

**Fig. 2:**
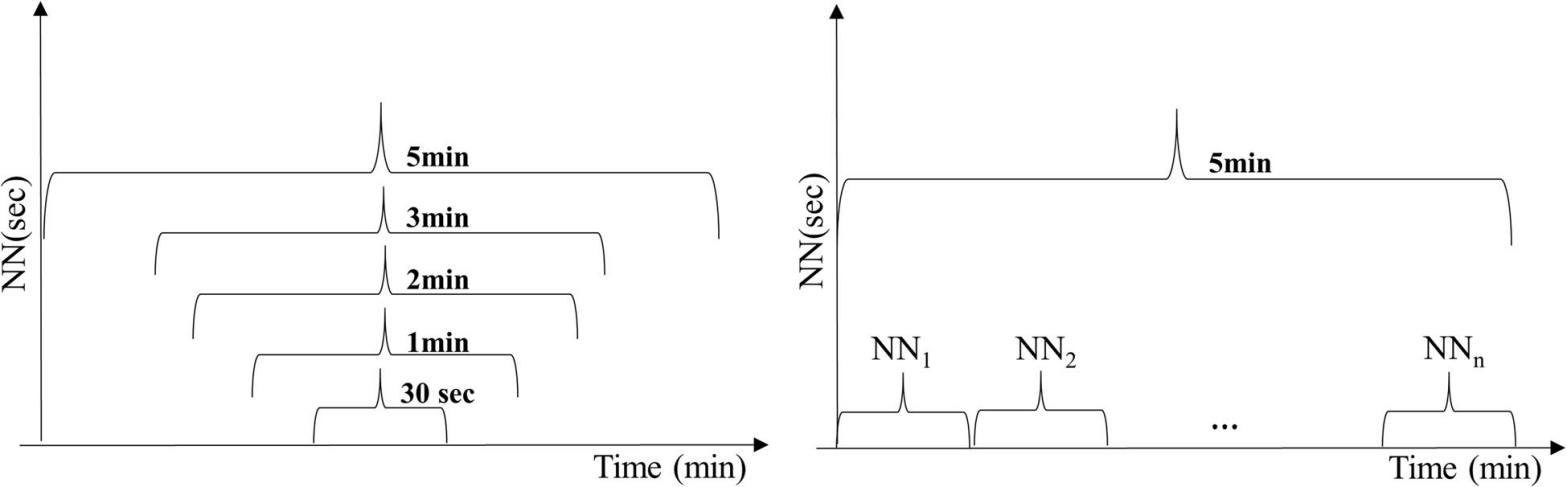
Segmentation process. The ultra-short HRV features were extracted from the central position of the 5 min NN excerpts (left-hand side). This procedure was repeated for the shortest significant length of NN excerpts. The shortest excerpts were extracted from different positions, without overlapping (right-hand side)

The same 5-min excerpts were later used to extract shorter NN excerpts (Fig. 2, left-hand side) from which the ultra-short HRV features were computed. The initial choice of extracting the central excerpts was arbitrary. Therefore, we decided to assess this choice by repeating the extraction from different locations within the 5-min excerpt (Fig. 2, right-hand side). This was done only with the shortest significant time length excerpt, resulting from the statistical significance and correlation analysis.

The HRV analysis was performed using Kubios software [51]. Time and frequency features were analyzed according to international guidelines [16], whereas non-linear features were analyzed as described in [7]. Spectral analysis can be performed by different methods, which are classified as non-parametric, such as the Fast Fourier Transform (FFT), and the autoregressive method (AR). The FFT has the advantage of the low computational cost. However, it has limitations such as a poor spectrum resolution, mainly when short data excerpts are used, and leakages. On the other hand, the AR method became popular because it produces a spectrum with better resolution when short data excerpts are used, and the spectrum can be divided into independent components. Therefore, frequency domain features were extracted from power spectrum estimated with autoregressive (AR) model methods. As reported in Additional file 1: Table S1^1^ 23 HRV features were extracted from 5-min, 3-min, 2-min, 1-min, and 30-s excerpts and subsequently analyzed.

However, some HRV features were not computable in ultra-short time excerpts. In fact, it is recommended that spectral analyses are performed on recordings at least 10 times longer than the wavelength of the lower frequency limit that is at least 2 min for the Low Frequency power (LF). Therefore, LF was not computed for excerpts below 2 min along with LF/HF ratio and total power. Additionally, High Frequency power (HF) was not computed for excerpts below 1 min [16]. As far as non-linear HRV features are concerned, less has been explored in the existing literature. However, approximate entropy (ApEn) values were excluded for length below 3 min, since they have shown to be unreliable due to the small number of samples presented in the RR series [31, 52, 53]. Moreover, when the length of the data was reduced to 30 s, most of the non-linear features became non-computable, due to the lack of samples. The entire list of features calculated for the different excerpt lengths is reported in Table 1.

**Table 1:** HRV feature trends

HRV Features	5 min	3 min	2 min	1 min	30 sec
MeanNN	↓↓	↓↓	↓↓	↓↓	↓↓
StdNN	↓↓	↓↓	↓↓	↓↓	↓↓
MeanHR	↑↑	↑↑	↑↑	↑↑	↑↑
Std HR	↑↑	↑↑	↑↑	↑↑	↑↑
RMSSD	↑	↑	↑	↑	↓
NN50	↑	↑	↑	↑	↑
pNN50	↓	↓	↑	↓	↓
LF	↓↓	↓↓	↓↓	–	–
HF	↓↓	↓↓	↓↓	↓↓	–
LF/HF	↓↓	↓↓	↓↓	–	–
TotPow	↓↓	↓↓	↓↓	–	–
SD1	↑	↑	↑	↑	↓
SD2	↓↓	↓↓	↓↓	↓↓	↓↓
ApEn	↓↓	↓↓	–	–	–
SampEn	↓↓	↓↓	↓↓	↓↓	–
D2	↓↓	↓↓	↓↓	↓↓	–
dfa1	↓↓	↓↓	↓↓	↓↓	–
dfa2	↑	↑	↑	↓	–
RPlmean	↑↑	↑↑	↑↑	↑	–
RPlmax	↓↓	↓↓	↓	↑	–
REC	↑↑	↑↑	↑↑	↑	–
RPadet	↑↑	↑	↑	↓	–
ShanEn	↑↑	↑↑	↑↑	↑↑	–

Trend ↓↓ (↑↑): significantly lower (higher) under stress (*p* < 0.05), ↓(↑) lower (higher) under stress (*p* > 0.05), − not computable

### Multiscale HRV comparison: Short vs ultra-short

**Fig. 3:**
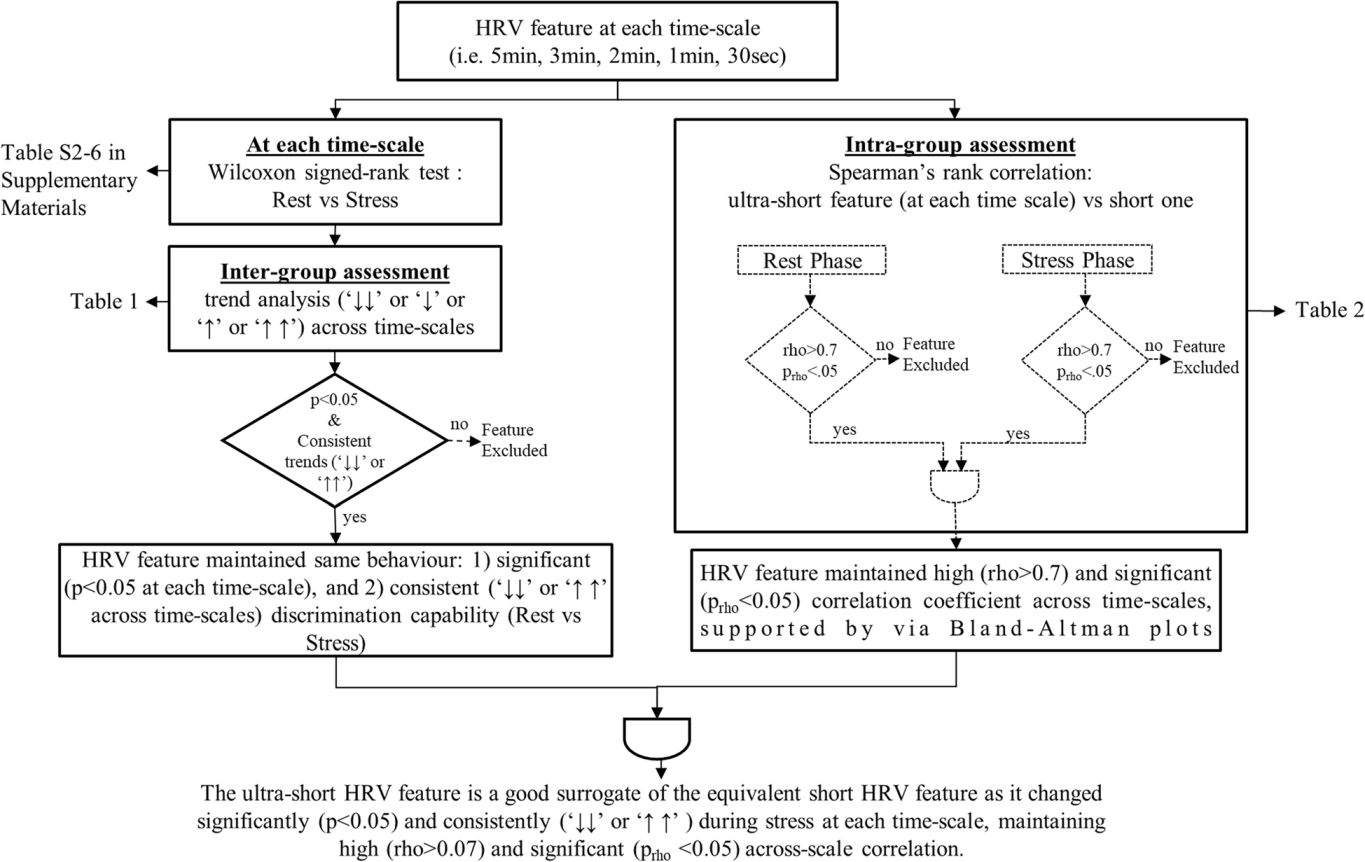
Methodological workflow for the identification of the good surrogates. This process was repeated for each HRV feature at each time scale. The complete list of feature computed at each time scale is reported in Table 1. p: *p*-value; trend analysis: ↓↓ (↑↑): significantly lower (higher) under stress (*p* < .05), ↓(↑) lower (higher) under stress (*p* > .05); rho: Spearman’s rank coefficient, prho: Spearman’s rank *p*-value

Median (MD), standard deviation (SD), 25th and 75th percentiles were calculated for all of the subjects to describe the distribution of HRV features during rest and stress phases at 5 min, 3 min, 2 min, 1 min, and 30 s (see Additional file 1:Table S2-S6). Moreover, a non-parametric statistical significance test and a correlation analysis were performed in parallel, as shown in Fig. 3, in order to select the subset of ultra-short HRV features that were good surrogates of short HRV features.

#### Non-parametric statistical significance and inter-group assessment

The non-parametric Wilcoxon signed-rank test was used to investigate the statistical significances (*p*-value< 0.05) of the HRV feature variations between stress and rest phases for each excerpt length (i.e., 5 min, 3 min, 2 min, 1 min, and 30 s).

The increase or decrease of the HRV feature median between rest and stress phases was reported, referred as median trend or feature trend, using the following convention [4, 54, 55]:

- Two arrows, ↓↓ (or ↑↑) were used to report a significant (*p*-value< 0.05) decrease (or increase) of a feature median during the stress phase;
- One arrow was used for non-significant variations: ↓ (or ↑) indicated a non-significant (p-value> 0.05) decrease (or increase) of a feature median during the stress phase.

Trend analysis consisted in inspecting inter-group changes (i.e., increase or decrease) of HRV feature median across time scales.

An HRV feature was assumed to maintain the same behavior across the 5 different time-scales (5 min, 3 min, 2 min, 1 min, and 30 s) if:

- the Wilcoxon’s test *p*-value was less than 0.05 between rest and stress phases over all time scales;
- the ultra-short HRV feature’s median trend was changing between rest and stress phases consistently with the equivalent short HRV feature.

#### Correlation analysis and Bland-Altman plots as intra-group assessment

An intra-group assessment was carried out using the Spearman’s rank correlation and Bland-Altman plots to investigate to what extent an ultra-short HRV feature was correlated with the equivalent short HRV feature during rest and stress conditions. For instances, MeanNN_3min_ (i.e., computed at 3 min during rest) VS MeanNN_5min_ (i.e., computed at 5 min during rest).

The statistical significance of this association was demonstrated by a p-value (p_rho_) lower than 0.05. As first screening, each ultra-short HRV feature was investigated against the equivalent short HRV feature during resting condition (Fig. 3). In addition, each ultra-short HRV feature was also explored during stress condition. However, a correlation coefficient is blind to the possibility of bias caused by the difference in the mean or standard deviation between two measurements; in other words, a strong correlation does not necessarily imply a close agreement. Therefore, Bland-Altman procedure was used to calculate 95% LoA (Limits of Agreement) [56]. In contrast to the traditional Bland-Altman plots, the measurements of the 5 min was plotted on the x-axis [39]. The bias was calculated as the median difference between the HRV features at 5 min and the ultra-short HRV features.

#### Surrogate feature subset selection

At this stage, it was assumed that an ultra-short HRV feature was a valid surrogate of the equivalent short one, only if:

- the feature maintained the same behavior between rest and stress conditions at each time scale;
- the ultra-short HRV feature was highly and significantly correlated (i.e. rho> 0.7 and p_rho_ < 0.05), with the equivalent short feature, over all of the time scales in both rest and stress phases.

### Classification and performance evaluation

Short HRV features (benchmark) were used to train, validate and test an automatic classifier to detect mental stress. The performance of this classifier was then tested inputting ultra-short HRV features in order to assess the discriminant power of ultra-short term HRV analysis.

To reduce overfitting problems and bias in the overall accuracy of the classifier, the whole dataset was randomly split per subject into two folders: folder 1 (60%) was used for feature selection, training and validation of the classifiers; folder 2 (40%) for testing the classifier. The reasoning behind this split is that a classifier should be tested on a set of data that is independent of the training data [55, 57]. Moreover, although the best approach is to select the minimum set of features using a different folder from the one adopted to train the machine learning classifier [55, 57], due to the small number of subjects, feature selection, training and validation were performed on the same folder (folder 1).

#### HRV feature selection for modelling

In order to minimize the over-fitting risk in a machine learning model, the number of features used in the model and its cardinality should be limited by the number of subjects presenting the event to detect (i.e., stress) in each folder [57]. Moreover, a significant small set of clinical features strongly simplifies the physiological interpretation of results, by directing attention only on the most informative features [57]. In addition, although the best approach is to select the minimum set of features using a different folder from the one adopted to train the machine learning model [55], due to the small number of subjects in this study, feature selection and model training were performed on the same folder (folder 1: 25 subjects). Because 5 min is defined as standard length for short term HRV analysis, the feature selection was performed using 5-min HRV features. Among the 23 HRV features initially computed, only those that showed to be good surrogates in the ultra-short time excerpts entered the feature selection process to build a classifier.

**Fig. 4:**
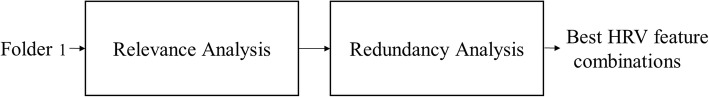
Framework of feature selection

The feature selection was based on two main stages (Fig. 4): the relevance analysis and the redundancy analysis. The former was performed using the Wilcoxon signed-rank test. The latter consisted in selecting only one feature from each cluster of features mutually correlated using Spearman’s rank correlation to reduce multicollinearity in the models. More details can be found in [55].

#### Machine learning methods

Five different machine-learning methods were used to develop classifiers aiming to automatically detect mental stress based on short HRV features: Support Vector Machine (SVM), which belongs to a general field of kernel-based machine learning methods and are used to efficiently classify both linearly and non-linearly separable data [58]; Multilayer Perceptron (MLP) consists of an artificial neural network of nodes (processing elements) arranged in layers [59]; Neighbor Search (IBK), which finds a group of K objects in the training set that are the closest to the test object, bases the assignment of a label on the predominance of a particular class in the neighborhood [60]; C4.5 builds decision trees from a set of training data, using the concept of information entropy [61]; Linear Discriminant Analysis (LDA) aims to find linear combinations of the input features that can provide an adequate separation between two classes [62].

Regarding model parameters, for the MLP classifier, the learning rate was varied from 0.3 to 0.9, the momentum from 0.2 to 1, the number of excerpts from 100 to 2000 and the number of hidden layers was set to 1 with 3 units [63, 64]; for the SVM, polynomial kernel function was used, varying the degree from 1 to 5. As regards IBK, it was trained with K equals to 1, 3 and 5 due to the binary nature of the classification problem [65]. C4.5 trees were developed by varying confidence factor (CF) for pruning from 0.05 to 0.5, minimum number of instances (ML) per leaf from 2 to 20. The algorithm parameters were tuned during training on folder 1.

Each of those methods was used with all of the combinations of relevant and non-redundant HRV features.

The Weka platform for knowledge discovery (version 3.6.10) (University of Waikato) was used to train, validate and test the classification models. This platform was issued as an open source software under the GNU General Public License [63].

#### Training, validation and testing

The training of the machine-learning models (including feature selection and algorithm parameter tuning) was performed on the folder 1 (25 subjects) and using 5-min HRV features (benchmark). Folder 1 was also used to validate the classifier using a k-fold cross-validation technique. The choice of the k-value is crucial in order to achieve high overall accuracy and reduce bias in the model. As a rule of thumb, the k-value should allow each of the k-folds to have at least 10 occurrences presenting the events to detect. Therefore, the 3-fold person-independent cross-validation approach was used to validate the models in folder 1 [55].

Binary classification performance measures were adopted according to the standard formulae reported in [66, 67].

Among the five different machine-learning methods used to train, validate and test the classifiers (SVM, MLP, IBK, C4.5, and LDA), the best-performing model was chosen as the classifier achieving the highest Area under the Curve (AUC), which is a reliable estimator of both sensitivity and specificity rates.

The model was then tested on folder 2 (17 subjects) using the short and ultra-short HRV features, in order to assess their efficacy in automatically detecting mental stress. Binary performances were computed again, but this time to observe how the performance of the model changed across different time-scales.

## Results

ECGs recorded from 42 healthy subjects (19 female, 23 male) were analyzed in the current study. Subjects were aged 18 to 25 years old (age: 21.5 ± 3.5), were no obese (BMI 22.3 ± 2.7) and were not taking any medication for the duration of the study. HRV features median (MD), standard deviation (SD), 25th and 75th percentiles calculated on 5-min, 3-min, 2-min, 1-min, and 30-s NN data series are given in the Additional file 1: Table S2-S6, respectively.^2^

### Multiscale HRV comparison: Short vs ultra-short

Table 1 summarizes the results of the significance and trend analysis, presenting the HRV features’ median trend at each time-scale. Table 1 also reports the HRV features calculated for the different excerpt lengths (i.e., features indicated with ‘-’ were not computable).

As shown in Table 1, from 5-min excerpts of NN data series, 18 out of the 23 selected HRV features showed significant changes from resting to stress conditions. Twelve out of these 18 features decreased significantly during stress phase, while the remaining 6 features (MeanHR, StdHR, RPlmean, REC, RPadet and ShanEn) showed a significant increase.

The second column in Table 1 demonstrates that from 3-min excerpts of NN data series all of the 23 features were computable, and 12 features decreased significantly during stress, while 5 (MeanHR, StdHR, RPlmean, REC, and ShanEn) increased significantly. However, RPladet which showed significant increase during 5 min, failed to show any significant change when the data length was shortened below 5 min.

The changes in the features extracted from 2-min excerpts, shown in the third column of Table 1, present the same significant trends as the 3-min features, apart from ApEn, which is not computable, and RPlmax, which is no longer significant (*p*-value< 0.05).

The changes in the features extracted from 1-min excerpts, shown in the fourth column of Table 1, present the same significant trends as the 2-min features, except for 3 HRV features (LF, LF/HF ratio, TotPow), which are not computable, and 2 HRV features (RPlmean and REC), which are no more significant (p-value< 0.05).

The changes in the features extracted from 30-s excerpts, shown in the fifth column of Table 1, present the same significant trends as the 1-min features, apart from those features that are not computable.

Table 2 shows the results of the correlation analysis used to select the subset of ultra-short HRV features that were good surrogates of short HRV features. The correlation analysis was run between ultra-short HRV features and the equivalent short ones. This analysis was not used to eliminate multicollinearity between features but to investigate the interdependence between an ultra-short HRV feature and its equivalent in 5-min excerpt.

**Table 2:** Correlation analysis of ultra-short HRV features vs equivalent short ones

	Rest Phase				Stress Phase			
HRV Features	3 vs 5 min	2 vs 5 min	1 vs 5 min	30 s vs 5 min	3 vs 5 min	2 vs 5 min	1 vs 5 min	30 s vs 5 min
**MeanNN**	**0.984**	**0.890**	**0.975**	**0.936**	**0.985**	**0.937**	**0.955**	**0.964**
**StdNN**	**0.954**	**0.875**	**0.905**	**0.749**	**0.962**	**0.912**	**0.791**	0.640
**MeanHR**	**0.984**	**0.891**	**0.975**	**0.947**	**0.985**	**0.938**	**0.954**	**0.964**
**StdHR**	**0.914**	**0.789**	**0.796**	0.635	**0.971**	**0.904**	**0.784**	0.696
**RMSSD**	**0.961**	**0.914**	**0.946**	**0.859**	**0.983**	**0.928**	**0.915**	**0.852**
**NN50**	**0.972**	**0.883**	**0.949**	**0.822**	**0.971**	**0.920**	**0.905**	**0.894**
**pNN50**	**0.967**	**0.882**	**0.943**	**0.818**	**0.969**	**0.915**	**0.913**	**0.881**
**LF**	**0.894**	**0.886**	–	–	**0.921**	**0.916**	–	–
**HF**	**0.915**	**0.906**	**0.901**	–	**0.925**	**0.915**	**0.798**	–
**LF/HF**	**0.830**	**0.839**	–	–	**0.846**	**0.807**	–	–
**TotPow**	**0.897**	**0.882**	–	–	**0.900**	**0.905**	–	–
**SD1**	**0.961**	**0.914**	**0.945**	**0.862**	**0.983**	**0.928**	**0.915**	**0.852**
**SD2**	**0.956**	**0.865**	**0.876**	**0.707**	**0.941**	**0.898**	**0.755**	0.694
**ApEn**	**0.771**	0.169	–	–	**0.918**	**0.790**	–	–
**SampEn**	**0.855**	0.666	0.681	–	**0.931**	**0.826**	0.599	–
**D2**	**0.922**	0.674	0.330	–	**0.967**	**0.876**	**0.816**	–
**dfa1**	0.661	0.687	0.637	–	**0.927**	**0.908**	**0.799**	–
**dfa2**	0.633	0.611	0.673	–	**0.767**	0.563	0.485	–
**RPlmean**	**0.837**	**0.708**	0.645	–	**0.901**	**0.730**	0.503	–
**RPlmax**	**0.738**	0.588	0.583	–	**0.896**	**0.737**	0.678	–
**REC**	**0.880**	0.643	0.608	–	**0.892**	0.689	0.513	–
**RPadet**	**0.852**	0.645	0.495	–	**0.948**	**0.817**	0.642	–
**ShanEn**	**0.795**	0.661	0.614	–	**0.907**	**0.720**	0.463	–

All the correlations resulted significant (p_rho_ < 0.05); in bold Spearman’s correlation coefficient (rho) greater than 0.7; −: not computable

Time domain HRV features maintained a significantly high correlation coefficient at 3 min, 2 min, and 1 min. Conversely, from 30-s excerpts, StdNN showed a Spearman coefficient above 0.70 at rest and below 0.70 during stress, while StdHR showed a Spearman coefficient below 0.70 during both rest and stress phases. Regarding frequency-domain HRV features, they showed to be highly correlated with the equivalent short HRV features at each time-scale (i.e., from 3 min to 1 min) during both resting and stress phases. As far as non-linear features are concerned, SD1 maintained a constant behavior between short and ultra-short term during rest and stress phases while SD2 was less correlated at 30 s during stress. ApEn, SampEn, D2, RPlmean, RPlmax, REC, RPadet and ShanEn showed to be highly correlated with short HRV features over 3-min excerpts during resting and stress conditions, while they resulted less correlated in shorter time-scales. In general, HRV features resulted less correlated in resting than during stress conditions. This is most likely due to the fact that HRV showed a more depressed dynamic during stress phase. Similar behaviors have been observed in other studies [68].

Due to this first analysis, the HRV features computed on 30-s excerpts were at this point excluded from the rest of the study due to the low number of HRV features behaving coherently with the benchmark. The results from the correlation analysis were supported by the visual inspection of the Bland-Altman plots. A decrease in bias and in width of the 95% LoA was observed as the excerpts length increased for all of the HRV features. A representative example is shown in Additional file 2: Figure S1 and Additional file 3: Figure S2.

As a result, MeanNN, StdNN, MeanHR, StdHR, HF and SD2 were selected as valid surrogates of short HRV features to investigate mental stress, as they displayed consistency across all of the excerpt lengths (i.e., from 5 to 1 min). Moreover, the discrimination power to automatically detect stress of these features across all of the excerpt lengths (i.e., from 5 to 1 min) was also corroborated as detailed in the section below.

### Classification and performance measurement

Regarding the feature selection process, all of the six HRV features (MeanNN, StdNN, MeanHR, StdHR, HF, and SD2), selected as valid surrogates of short HRV features resulted also relevant in folder 1. This was not a trivial result given the lower number of subjects included in folder 1. In fact, a reduction in the number of subjects may result in an increase of *p*-values. As result of the redundancy analysis the minimum set of relevant but mutually non-correlated features resulted to be: MeanNN, StdHR, and HF.

Each machine learning method was trained and validated with this combination of short HRV features (MeanNN, StdHR, and HF) using folder 1. The classifiers were then tested on short HRV features using folder 2 as shown in Table 3.

**Table 3:** Model performance measurements estimated on the test set (Folder 2) on 5 min excerpts

Method	Parameters	AUC	SEN	SPE	ACC
MLP	LR = 0.3; ML = 0.2; NE = 500	98%	100%	88%	94%
SVM	PolyKernel, E = 1.0	88%	88%	88%	88%
C4.5	CF = 0.25; ML = 2	94%	88%	100%	94%
IBK	K = 3	99%	88%	100%	94%
LDA	**–**	98%	88%	100%	94%

*MLP* Multilayer Perceptron, *SVM* Support Vector Machine, *C4.5* decision trees, *IBK* Neighbor Search, *LDA* Linear Discriminate Analysis, *AUC* area under the curve, *SEN* sensitivity, *SPE* specificity, *ACC* accuracy

According to the criteria defined above, the IBK classifier showed the highest AUC with 88% sensitivity, 100% specificity, 94% accuracy, and 99% AUC, using MeanNN, StdHR and HF as HRV features. Therefore, the IBK was chosen as model to automatically detect mental stress.

The IBK model was then tested using ultra-short HRV features in folder 2 to further evaluate their capability to automatically detect mental stress (Table 4).

**Table 4:** Model performance measurements on different time-scale excerpts

Duration	AUC	SEN	SPE	ACC
3 min	97%	94%	94%	94%
2 min	93%	94%	88%	91%
1 min	93%	82%	94%	88%

*AUC* area under the curve, *SEN* sensitivity, *SPE* specificity, *ACC* accuracy

The length of data seemed to slightly affect the performance of the model. However, as shown in Table 4, the model outperformed in 3-min time-scale with 97% AUC.

Compared to the short term performances, sensitivity increased by 6% and specificity decreased by 6% respectively using 3-min excerpts. Nevertheless, the model achieved good performances also using 1-min HRV excerpts. After observing these results, the model was also assessed on consecutive 1-min excerpts (as shown in Fig. 2, right-hand side) within the 5-min NN data series in order to understand if the performances were changing significantly according to the extracted excerpts. In fact, the performances using 1-min HRV features showed to be constantly good with 86 ± 4.1% sensitivity, 95 ± 4.4% specificity and 92 ± 3.75% accuracy.

## Discussion

The current study aimed to investigate if ultra-short HRV features are valid surrogates of short ones to automatically detect mental stress. This is a topic of growing interest. In fact, the continued rise of consumer wearable devices able to instantaneously assess mental stress level is raising the attention of the scientific community around the use of HRV features computed over excerpts shorter than 5 min [22].

Differently from Melillo et al. [7], this study explored the validity of ultra-short HRV features as surrogates of short HRV features to detect mental stress in real-life scenario. Moreover, in Melillo et al. [7] only non-linear HRV features were analyzed in 5-min excerpts and employed to develop a linear classifier.

Regarding the methodology, this study presents an innovative method to assess the minimum length of HRV excerpts to detect mental stress in healthy young subjects. In fact, to the best of the authors’ knowledge [22], only two studies evaluated the reliability of ultra-short HRV features during stress condition, but only using statistical significance tests, which as demonstrated in this study are not sufficient to draw any conclusion [31, 69]. In fact, differently from the methods described in the current paper, Pereira et al. used only a parametric statistical test (one-way ANOVA) to determine which HRV features (i.e., 220, 150, 100 and 50 s) could discriminate between rest and stress sessions (*p* < 0.05) with small windows of analysis [69]. For their part, Salahuddin et al. used the non-parametric Kruskal–Wallis test to assess that ultra-short term analysis was not significantly different to the short term analysis if the *p*-value was greater than 0.05 and Wilcoxon sign-rank test (p < 0.05) to find the shortest duration that distinguished between rest and stress phases. However, no correlation or machine learning methods were utilized to validate their findings. Moreover, if the p-value is greater than 0.05 then the null hypothesis cannot be neither rejected nor accepted [70]. Therefore, no conclusion can be drawn using only the statistical significance tests, which make the results reported in [31] not sufficiently reliable. Hence, it is difficult to compare the results reported in [31] with the one reported in the current paper. Unfortunately, this study has been used to support the majority of works related to mental stress detection using ultra-short HRV features [31]. In fact, many wearable systems [28, 41, 42] and scientific studies [26–28, 30, 32, 41, 42] monitoring stress via ultra-short term HRV analysis have based their feature selection on Salahuddin et al. [31] results, which should be read more carefully.

Other studies have investigated the reliability and accuracy of ultra-short HRV features in different conditions (e.g., athletic performance, acoustic sounds and controlled resting conditions) [33–36, 38, 39]. However, none of these studies employed rigorous statistical approaches to identify ultra-short HRV features as good surrogates of short term ones [22].

Differently from our study, few studies only employed correlation tests to prove that ultra-short term HRV features behaved as good surrogates of short-term ones, concluding that ultra-short HRV features were good surrogates of short-term ones if significantly correlated with their equivalent short HRV features [34, 35, 38]. This result is arguable because, as demonstrated in our study, although an appropriate correlation test is the first step for the identification of a good surrogate, a much stronger condition than correlation is required to identify a surrogate [22, 71].

Other studies performed both statistical significance test and correlation analysis in alignment with our study, but they presented various methodological ambiguities [33, 36, 39]. For more details refer to [22]. Only one study investigated HRV features in time domain in 10, 30 and 120 s compared to 5 min, using a more rigorous method [39]. In fact, they used Pearson correlation, after log transforming HRV features, Bland-Altman plots and Cohen’s d. However, although the approach used in Munoz et al. [39] to assess the validity of ultra-short HRV features seems more rigorous than other studies [22], they only investigated 2 time domain HRV features (SDNN and RMSSD) in resting condition.

Regarding our results, the statistical analysis in the short term showed a significant depressed HRV during stress, in agreement with the previously published literature [4]. Ultra-short term HRV features also resulted in being significantly depressed during mental stress over each time-scale. Concerning the HRV features in time domain, all of them maintain the same behavior across the 5 different time-scales (i.e., 5 min, 3 min, 2 min, 1 min, and 30 s). Moreover, four of them (MeanNN, StdNN, MeanHR and StdHR) were also significantly different between rest and stress phases and were significantly correlated (Spearman’s rank rho> 0.7) across time-scales (i.e., each ultra-short vs short time-scale per each feature). These results, achieved with a more robust method, confirm the findings of Baek et al. [36], McNames and Aboy [35], Nussinovitch et al. [34], Pereira et al. [69] and Munoz et al. [39], which showed that MeanNN, StdNN, MeanHR are reliable for length from 5 to 1 min in a controlled resting condition. However, some HRV features that showed to be good surrogates in the existing literature, failed to show good results in the present study. Our interpretation of this result is that the method used in the present study is based on more stringent and reliable requirements, compared to other studies, which demonstrated significant methodological limitations [22]. Concerning the HRV features in frequency domain, it is well-known that a minimum of 1 min is required to estimate HF and a minimum of 2 min is required to estimate LF component [16, 36]. Accordingly, the present study showed that for HRV features in frequency domain such as LF, the minimum length is 2 min. However, HF component could be extracted from 1-min excerpts, as confirmed by the fact that in this study HF resulted to be a good surrogate of the 5 min equivalent. In fact, as also proved by Baek et al. [36], LF had a very low Pearson coefficient below 2 min whilst HF below 1 min. In relation to non-linear HRV features, no study has investigated their reliability in excerpts shorter than 5 min. The current study empirically demonstrated that they lose their utility for excerpts below 3 min due to computational problems. In fact, non-linear HRV features require a high number of samples in order to appreciate the dynamics of the heartbeat series over time. Only two HRV non-linear features (SD1 and SD2) showed to be good surrogates over 3, 2 and 1-min lengths as also shown by Nardelli et al. [38].

Although our study employed only 42 healthy subjects to develop a model to automatically detect stress, it is able to detect stress with higher accuracy than the models presented in the existing literature [7, 15, 20, 23, 24, 27–29, 32, 72].

Three studies proposed a model to detect mental stress using short term HRV analysis [7, 20, 72], whilst seven studies developed a model for the detection of mental stress using ultra-short HRV features [15, 23, 24, 27–29, 32]. Melillo et al. [7] adopted the same dataset as in this study and proposed a model based on LDA, employing only three HRV non-linear features: SD1, SD2 and ApEn in short term HRV analysis. The model proposed in their study, achieved sensitivity, specificity and accuracy, of 86, 95 and 90%, respectively, which are lower than the ones achieved by the model developed in this study. Whereas Traina et al. [72] studied the Pearson correlation between frequency domain measures before and after the stress session, demonstrating that those correlations were significant. However, as discussed above, the Pearson correlation lays on the assumption that the HRV measures are normally distributed, yet HRV frequency measures are not. In 2015, Munla et al. [20] used an SVM-RBF classifier using time and non-linear HRV features, with only 16 different individuals, to predict drivers’ stress with an accuracy of 83%. However, no validation or testing was applied in that study.

Mayya et al. [28] proposed a method for automatically detecting mental stress using smartphone and focusing on 1-min HRV features. The model was built on the assumption that ultra-short HRV features were relevant according to the available literature [31], which has been proved to lack of a robust method to identify ultra-short HRV features that are good surrogates of short HRV features [22]. They used a multinomial logistic regression applied to 2 features, RMSSD and dfa1, which were excluded in our study, and achieved 80.5% accuracy, which is lower than the accuracy achieved in the present study, supporting the idea that an suboptimal ultra-short feature selection generates low performances. Choi et al. [24], Brisinda et al. [27] and Sun et al. [32] also proposed a method to automatically detect mental stress focusing on 4-min, 2-min and 1-min HRV features respectively. Also in these studies, the models were built on the assumption that ultra-short HRV features were relevant according to the available literature, although Brisinda et al. [27] confirmed their findings using only ICC analysis. These studies used linear classifiers achieving accuracy lower than the one achieved in the current study. Other models were developed using ultra-short term HRV analysis along with other physiological measurements but they are not discussed here [15, 29, 32]. To conclude, none of those papers achieved better results than the one presented in this study. This also supports our convincement that a reliable identification of good surrogates is important to identify a good set of features aiming to detect mental stress. However, it is important to highlight that these studies employed protocols and sample sizes different from our study and therefore, a strict comparison of the classifiers’ performance may be equivocal [15, 23, 24, 27–29, 32].

The current study showed that IBK was able to detect stressed subjects with 88, 100, 94% of sensitivity, specificity and accuracy respectively, using short HRV features (MeanNN, StdHR and HF). IBK was the most recurrent machine learning used among the papers identified in the existing literature [23, 24, 29].

Finally, it is useful to mention that the proposed methodology could be used in any application aiming to automatically detect a condition using ultra-short HRV features. In particular, the proposed method can improve the identification of the minimal length of HRV excerpts enabling the detection of an anomaly in real time.

## Conclusion

Currently, 5-min recordings are regarded as being an appropriate option for HRV analysis to detect mental stress in healthy subjects. However, the continued rise in the interest of everyday wearable devices being able to instantaneously assess mental stress level is rising the attention of the scientific community around the use of RR interval shorter than 5 min.

This study demonstrates that not all the ultra-short HRV features are good surrogates of short term ones. In fact, only six ultra-short HRV features resulted to be good surrogates of short term ones: MeanNN, StdNN, MeanHR, StdHR, HF, and SD2. Those six features displayed consistency across all the excerpt lengths (i.e., from 5 to 1 min) and MeanNN, StdHR and HF showed good performance if employed in a well-dimensioned automatic classifier.

Moreover, an automatic classifier based on IBK is able to detect stressed subjects with very high performances, using 3-min HRV analysis, and relatively good performances using 1-min HRV excerpts. The former achieved sensitivity, specificity and accuracy of 94, 94 and 94% respectively and the latter achieved sensitivity, specificity and accuracy of 82, 94 and 88% respectively.

Therefore, we conclude that it is possible to automatically detect mental stress using ultra-short HRV features with excerpts not shorter than 1 min. According to the specific application, 3- or 2-min excerpts could be preferable, because features having a clear physiological significance (e.g., HF and LF) remain computable.

## Additional files

Additional file 1: **Table S1-S6.** HRV features and their distributions. HRV feature distributions at rest and stress and different time scales (i.e. 5 min, 3 min, 2 min, 1 min, and 30s). (PDF 307 kb)

Additional file 2: **Figure S1.** Bland-Altman Plot of MeanNN during Rest. Bland-Altman Plot of MeanNN during Rest. (PDF 136 kb)

Additional file 3: **Figure S2.** Bland-Altman Plot of MeanNN during Stress. Bland-Altman Plot of MeanNN during Stress. (PDF 207 kb)

## Abbreviations

ACC: Accuracy
AUC: Area under the curve
C4.5: Decision trees classifier
ECG: Electrocardiogram
HRV: Heart rate variability
IBK: K-nearest neighbors classifier
LDA: Linear discriminant analysis classifier
MLP: Multilayer perceptron classifier
Rho: Spearman’s correlation coefficient
SEN: Sensitivity
SPE: Specificity
SVM: Support vector machine classifier

## Footnotes

1 Supplementary materials are available in the supporting document

2 Supplementary materials are available in the supporting document
