# Supplementary material for: Ultra-short term HRV features as surrogates of short term HRV: a case study on mental stress detection in real life
Source: BMC Med Inform Decis Mak. 2019 Jan 17;19:12. doi: 10.1186/s12911-019-0742-y (PMC6335694; doi:10.1186/s12911-019-0742-y)
Supplement: Supplementary file 1 — Table S1-S6. HRV features and their distributions. HRV feature distributions at rest and stress and different time scales (i.e. 5 min, 3 min, 2 min, 1 min, and 30s). (PDF 307 kb) [file 12911_2019_742_MOESM1_ESM.pdf]

SUPPLEMENTARY MATERIALS

HRV Features analysed in the current study are reported in Table S1.

TABLE S1
HRV FEATURES

HRV Measures	Units	Description
**Time Domain**		
**MeanNN**	[ms]	The mean of NN intervals
**StdNN**	[ms]	Standard deviation of NN intervals
**MeanHR**	[1/min]	The mean heart rate
**StdHR**	1/min]	Standard deviation of instantaneous heart rate values
**RMSSD**	[ms]	Square root of the mean squared differences between successive NN intervals
**NN50**	-	Number of successive NN interval pairs that differ more than 50 ms
**pNN50**	[%]	NN50 divided by the total number of NN intervals
**Frequency Domain**		
**LF**	[ms^2]	Low Frequency power (0.04-0.15Hz)
**HF**	[ms^2]	High Frequency power
**LF/HF**	-	Ratio between LF and HF band powers
**TotPow**	[ms^2]	Total power
**Non Linear Domain**		
**SD1, SD2**	[ms]	The standard deviation of the Poincare' plot perpendicular to (SD1) and along (SD2) the line-of-identity
**ApEn**	-	Approximate entropy
**SampEn**	-	Sample entropy
**D2**	-	Correlation dimension
**dfa1, dfa2**	-	Detrended fluctuation analysis: Short term and Long term fluctuation slope
**RPlmean**	[beats]	Recurrence plot analysis: Mean line length
**RPlmax**	[beats]	Recurrence plot analysis: Maximum line length
**REC**	[%]	Recurrence rate
**RPadet**	[%]	Recurrence plot analysis: Determinism
**ShanEn**	-	Shannon entropy

HRV features median (MD), standard deviation (SD), 25th and 75th percentiles extracted from 5 min, 3 min, 2 min, 1 min and 30 sec NN data series in S2, S3, S4, S5 and S6 respectively

TABLE S2

HRV FEATURES IN REST AND STRESS FROM 5 MIN NN DATA SERIES

Short term: 5 min										
	Rest				Stress					
**HRV features**	**MD**	**SD**	**25**	**75**	**MD**	**SD**	**25**	**75**	**p-value**	**Trend**
**MeanNN**	726.988	83.695	647.623	776.49	483.594	66.464	446.027	512.257	0.000	↓↓
**StdNN**	59.642	18.383	44.455	70.007	36.619	16.266	26.438	46.990	0.000	↓↓
**MeanHR**	82.976	9.704	78.038	93.138	125.242	16.711	117.422	135.807	0.000	↑↑
**StdHR**	6.378	1.812	5.579	7.234	9.084	3.350	6.997	11.509	0.000	↑↑
**RMSSD**	33.806	14.719	22.848	42.490	34.120	16.643	18.069	46.997	0.996	↑
**NN50**	49.500	41.888	17.000	80.000	55.000	68.568	12.000	102.000	0.499	↑
**pNN50**	12.332	11.336	3.899	19.722	9.218	10.004	1.984	17.354	0.341	↓
**LF**	1661.164	1252.052	995.772	2497.8	454.370	874.453	186.016	1070.183	0.000	↓↓
**HF**	381.739	488.299	212.089	591.26	141.648	233.871	67.719	344.740	0.000	↓↓
**LF/HF**	4.646	2.512	2.811	6.424	3.267	2.181	1.943	4.662	0.011	↓↓
**TotPow**	3313.660	2160.757	1953.027	4694.2	1045.958	1709.43	427.809	2214.643	0.000	↓↓
**SD1**	23.936	10.423	16.172	30.081	24.146	11.777	12.788	33.267	0.996	↑
**SD2**	79.555	24.420	61.047	94.592	46.969	22.878	31.713	57.903	0.000	↓↓
**ApEn**	1.103	0.125	1.020	1.193	0.933	0.240	0.842	1.178	0.012	↓↓
**SampEn**	1.325	0.272	1.111	1.546	0.876	0.392	0.760	1.228	0.000	↓↓
**D2**	3.179	1.090	2.245	3.544	1.496	1.283	0.469	2.575	0.000	↓↓
**dfa1**	1.439	0.161	1.283	1.511	1.043	0.446	0.690	1.447	0.000	↓↓
**dfa2**	0.716	0.183	0.644	0.954	0.767	0.136	0.679	0.852	0.862	↑
**RPlmean**	10.439	2.479	9.519	12.688	13.326	6.771	11.105	16.920	0.002	↑↑
**RPlmax**	282.000	111.223	178.000	384.00	179.000	136.599	86.000	282.000	0.004	↓↓
**REC**	32.570	6.276	29.553	37.592	43.252	12.050	36.107	49.023	0.000	↑↑
**RPadet**	98.776	0.858	98.314	99.204	99.254	1.277	98.138	99.633	0.034	↑↑
**ShanEn**	3.139	0.233	3.044	3.363	3.418	0.398	3.210	3.642	0.001	↑↑

MD.: Median; SD: Standard Deviation; Trend;↓↓ (↑↑): significantly lower (higher) under stress (p<.05); ↓(↑): lower (higher) under stress (p>.05)

TABLE S3

HRV FEATURES IN REST AND STRESS FROM 3 MIN NN DATA SERIES

Ultra-short term: 3 min										
	Rest				Stress					
**HRV features**	**MD**	**SD**	**25**	**75**	**MD**	**SD**	**25**	**75**	**p-value**	**Trend**
**MeanNN**	725.245	82.97562	651.83	773.57	482.705	67.07718	443.36	522.57	0.000	↓↓
**StdNN**	57.709	17.957	41.214	69.298	35.211	17.773	26.797	46.104	0.000	↓↓
**MeanHR**	83.367	9.644	78.045	92.336	124.860	17.098	117.070	136.07	0.000	↑↑
**StdHR**	6.251	1.866	5.397	7.449	8.943	3.488	6.452	12.57	0.000	↑↑
**RMSSD**	33.283	14.465	24.099	42.016	34.344	16.637	18.856	46.81	0.764	↑
**NN50**	28.500	24.984	10.000	44.000	35.500	40.741	7.000	66	0.359	↑
**pNN50**	11.941	11.087	3.831	19.731	9.968	9.954	1.728	17.5	0.418	↓
**LF**	1397.950	1475.536	569.630	2224.400	415.345	852.459	106.490	889.28	0.000	↓↓
**HF**	297.630	442.760	173.440	516.530	101.281	281.341	56.541	291.13	0.000	↓↓
**LF/HF**	4.597	2.746	2.912	5.674	3.607	3.353	1.553	5.1267	0.048	↓↓
**TotPow**	2529.000	2154.584	1845.000	4745.000	735.500	2056.264	286.000	1642	0.000	↓↓
**SD1**	23.583	10.253	17.081	29.774	24.317	11.779	13.349	33.147	0.785	↑
**SD2**	77.528	23.960	54.410	90.998	40.775	25.432	30.339	58.666	0.000	↓↓
**ApEn**	0.987	0.093	0.923	1.039	0.870	0.192	0.780	1.0491	0.041	↓↓
**SampEn**	1.350	0.258	1.173	1.531	0.928	0.420	0.701	1.2753	0.000	↓↓
**D2**	3.045	1.069	2.044	3.385	1.398	1.208	0.331	2.4175	0.000	↓↓
**dfa1**	1.440	0.190	1.302	1.543	1.075	0.468	0.653	1.3822	0.000	↓↓
**dfa2**	0.728	0.188	0.614	0.875	0.761	0.170	0.624	0.86762	0.911	↑
**RPlmean**	10.171	2.876	8.898	13.569	13.224	7.291	10.427	16.665	0.023	↑↑
**RPlmax**	179.000	65.203	110.000	230.000	141.000	96.181	74.000	201	0.117	↓↓
**REC**	33.058	7.586	27.676	38.662	42.708	14.129	32.762	50.409	0.003	↑↑
**RPadet**	98.774	0.935	98.097	99.249	99.195	1.541	97.826	99.632	0.074	↑
**ShanEn**	3.089	0.264	2.943	3.332	3.361	0.428	3.131	3.5907	0.009	↑↑

MD.: Median; SD: Standard Deviation; Trend;↓↓ (↑↑): significantly lower (higher) under stress ($p<.05$); ↓(↑): lower (higher) under stress ($p>.05$)

TABLE S4

HRV FEATURES IN REST AND STRESS FROM 2 MIN NN DATA SERIES

Ultra-short term: 2 min										
	Rest				Stress					
**HRV features**	**MD**	**SD**	**25**	**75**	**MD**	**SD**	**25**	**75**	**p-value**	**Trend**
**MeanNN**	720.278	97.448	651.239	767.654	477.222	69.265	440.625	528.335	0.000	↓↓
**StdNN**	49.102	19.016	39.252	69.378	34.953	18.357	26.144	43.930	0.000	↓↓
**MeanHR**	83.786	14.068	78.615	92.636	126.107	17.810	115.474	137.403	0.000	↑↑
**StdHR**	6.147	2.044	5.174	7.253	8.481	3.560	6.715	12.470	0.000	↑↑
**RMSSD**	32.819	14.947	20.201	39.421	34.791	17.349	17.536	47.076	0.986	↑
**NN50**	16.000	16.992	7.000	26.000	23.000	28.112	3.000	42.000	0.496	↑
**pNN50**	9.650	11.153	3.784	16.129	10.333	10.147	1.038	16.342	0.452	↑
**LF**	1359.861	1362.088	736.544	2559.886	416.867	997.892	145.482	836.823	0.000	↓↓
**HF**	312.068	443.887	165.133	564.119	134.815	280.732	61.680	264.583	0.000	↓↓
**LF/HF**	4.498	2.977	2.997	5.899	3.321	3.195	1.517	5.093	0.031	↓↓
**TotPow**	2460.120	2046.273	1637.191	4269.091	814.113	2180.514	305.468	1735.691	0.000	↓↓
**SD1**	23.275	10.610	14.323	27.963	24.651	12.291	12.430	33.350	1.000	↑
**SD2**	66.157	25.363	53.775	93.058	38.650	26.374	29.384	55.751	0.000	↓↓
**ApEn**	0.856	0.086	0.796	0.893	0.809	0.153	0.692	0.929	-	-
**SampEn**	1.311	0.337	1.128	1.502	0.952	0.405	0.663	1.210	0.000	↓↓
**D2**	2.603	1.018	1.798	3.148	1.187	1.241	0.320	2.765	0.002	↓↓
**dfa1**	1.419	0.219	1.346	1.590	0.988	0.482	0.656	1.542	0.000	↓↓
**dfa2**	0.651	0.220	0.572	0.864	0.690	0.213	0.592	0.847	0.549	↑
**RPlmean**	9.821	3.798	8.565	12.312	12.828	8.599	9.998	15.587	0.021	↑↑
**RPlmax**	135.000	48.793	82.000	156.000	109.000	72.475	62.000	181.000	0.734	↓
**REC**	31.491	8.596	26.695	38.403	40.450	15.503	31.421	49.258	0.011	↑↑
**RPadet**	98.780	1.003	98.046	99.207	99.154	1.797	98.224	99.685	0.089	↑
**ShanEn**	3.008	0.307	2.854	3.225	3.232	0.429	3.030	3.506	0.021	↑↑

MD.: Median; SD: Standard Deviation; Trend;↓↓ (↑↑): significantly lower (higher) under stress ($p<.05$); ↓(↑): lower (higher) under stress ($p>.05$); -: not computable

TABLE S5

HRV FEATURES IN REST AND STRESS FROM 1 MIN NN DATA SERIES

Ultra-short term: 1 min										
	Rest				Stress					
**HRV features**	**MD**	**SD**	**25**	**75**	**MD**	**SD**	**25**	**75**	**p-value**	**Trend**
**MeanNN**	725.169	87.204	658.022	777.922	492.167	78.866	450.060	539.117	0.000	↓↓
**StdNN**	48.554	18.505	40.710	63.065	33.978	17.420	25.249	43.979	0.000	↓↓
**MeanHR**	83.153	10.087	77.476	91.438	122.549	18.442	111.545	133.686	0.000	↑↑
**StdHR**	5.799	2.433	4.624	6.731	8.003	3.500	6.124	10.981	0.000	↑↑
**RMSSD**	30.806	15.435	25.018	38.449	31.269	18.370	13.395	45.394	0.823	↑
**NN50**	7.500	8.396	4.000	14.000	11.500	14.956	1.000	23.000	0.382	↑
**pNN50**	10.201	10.539	4.444	17.722	10.156	11.444	0.848	16.556	0.505	↓
**LF**	1605.850	1567.523	563.291	2711.665	284.957	828.234	112.012	725.210	-	-
**HF**	367.511	381.504	190.971	497.143	103.378	280.790	42.947	230.933	0.000	↓↓
**LF/HF**	4.398	3.216	2.906	5.730	3.443	4.408	1.427	6.237	-	-
**TotPow**	2557.366	2143.559	1464.690	4488.421	550.976	1614.007	212.826	1335.339	-	-
**SD1**	21.919	10.988	17.790	27.369	22.197	13.042	9.514	32.244	0.775	↑
**SD2**	66.060	24.540	55.730	85.111	36.032	24.563	27.547	53.614	0.000	↓↓
**ApEn**	0.602	0.085	0.545	0.655	0.629	0.098	0.584	0.696	-	-
**SampEn**	1.305	0.336	1.166	1.639	0.984	0.455	0.727	1.474	0.001	↓↓
**D2**	2.509	0.875	1.737	2.868	1.317	1.196	0.417	2.632	0.002	↓↓
**dfa1**	1.473	0.221	1.205	1.577	1.200	0.505	0.785	1.545	0.009	↓↓
**dfa2**	0.684	0.337	0.552	0.957	0.684	0.276	0.553	0.913	0.540	↓
**RPlmean**	8.410	2.493	7.388	9.519	10.558	4.509	7.426	13.639	0.075	↑
**RPlmax**	67.000	16.783	51.000	77.000	71.000	32.599	36.000	97.000	0.681	↑
**REC**	29.679	7.495	24.992	36.131	34.787	13.899	25.887	44.335	0.107	↑
**RPadet**	98.278	1.311	97.248	98.799	98.076	2.246	95.955	99.301	0.957	↓
**ShanEn**	2.659	0.293	2.471	2.864	2.933	0.372	2.623	3.151	0.005	↑↑

MD.: Median; SD: Standard Deviation; Trend;↓↓ (↑↑): significantly lower (higher) under stress (p<.05); ↓(↑): lower (higher) under stress (p>.05); -: not computable

TABLE S6

HRV FEATURES IN REST AND STRESS FROM 30 SEC NN DATA SERIES

Ultra-short term: 30 sec										
	Rest				Stress					
**HRV features**	**MD**	**SD**	**25**	**75**	**MD**	**SD**	**25**	**75**	**p-value**	**Trend**
**MeanNN**	720.853	89.963	650.870	772.564	480.738	72.726	444.000	529.298	0.000	↓↓
**StdNN**	48.619	18.875	33.701	66.484	31.894	18.916	19.215	41.428	0.000	↓↓
**MeanHR**	83.864	10.562	77.769	92.424	124.951	18.788	113.421	135.618	0.000	↑↑
**StdHR**	5.583	2.782	4.300	6.792	6.850	4.633	5.209	11.562	0.021	↑↑
**RMSSD**	30.829	17.345	21.260	42.370	28.998	21.791	10.732	47.465	0.247	↓
**NN50**	3.500	5.190	1.000	8.000	3.500	8.143	0.000	14.000	1.000	↑
**pNN50**	8.957	12.375	2.564	22.500	5.489	11.979	0.000	19.737	0.238	↓
**LF**	1207.038	1820.654	525.548	2956.652	180.888	1156.513	83.894	512.759	-	-
**HF**	242.283	928.954	142.342	453.410	69.902	214.315	31.608	157.711	-	-
**LF/HF**	5.292	5.237	1.760	8.531	3.699	7.685	1.320	7.962	-	-
**TotPow**	2051.232	7451.549	1058.810	3868.496	366.116	2403.753	162.112	1198.807	-	-
**SD1**	22.090	12.422	15.277	30.397	20.695	15.527	7.671	33.938	0.239	↓
**SD2**	64.777	25.347	43.553	89.512	34.382	25.793	22.181	47.197	0.000	↓↓

MD.: Median; SD: Standard Deviation; Trend;↓↓ (↑↑): significantly lower (higher) under stress (p<.05); ↓(↑): lower (higher) under stress (p>.05); -: not computable
