# Supplementary figures and images for: Ultra-short term HRV features as surrogates of short term HRV: a case study on mental stress detection in real life
Source: BMC Med Inform Decis Mak. 2019 Jan 17;19:12. doi: 10.1186/s12911-019-0742-y (PMC6335694; doi:10.1186/s12911-019-0742-y)

## MeanNN during Rest

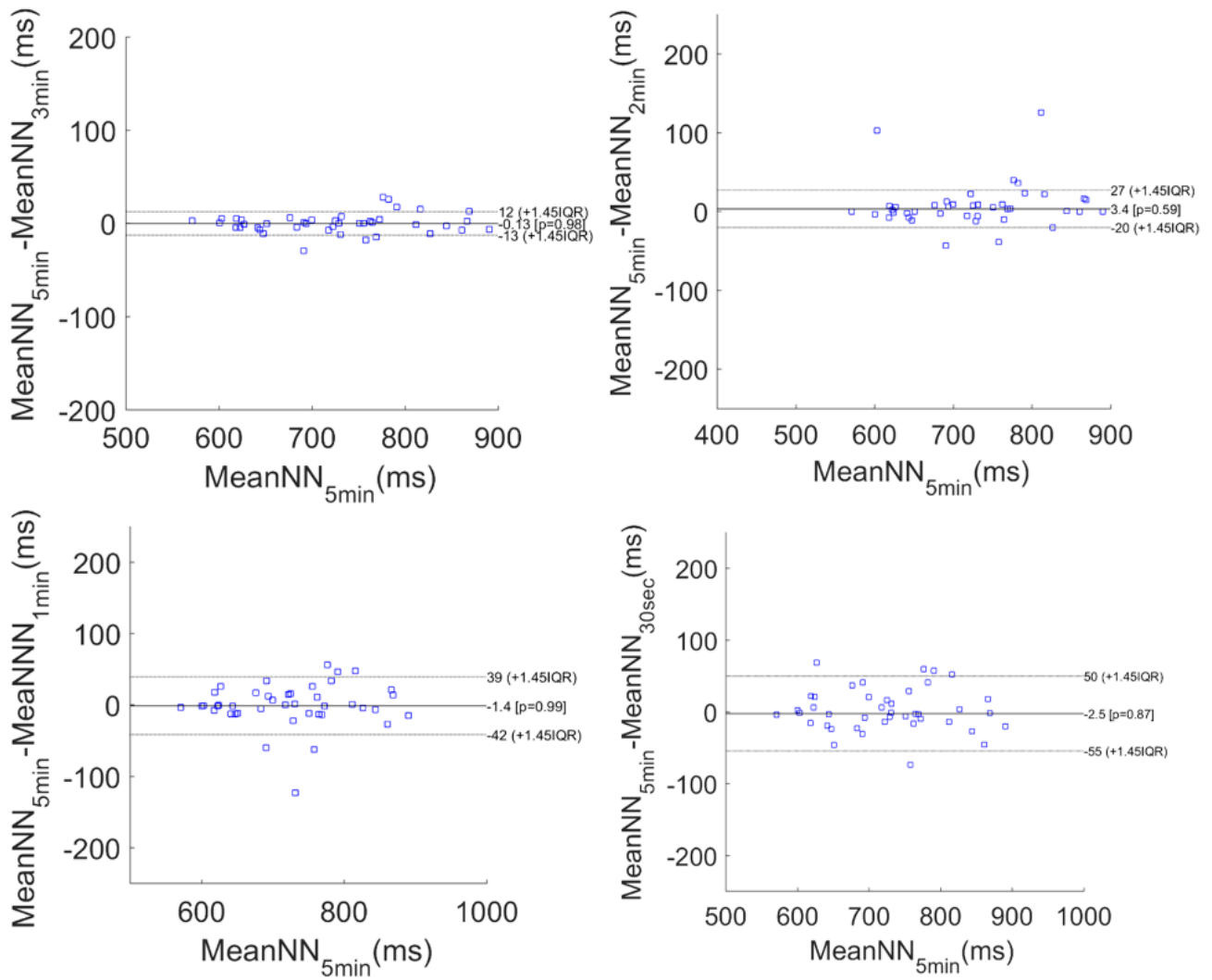

Fig. S1 Bland-Altman Plot of MeanNN during Rest. IQR: Interquartile range.

Supplement: Supplementary file 2 — Figure S1. Bland-Altman Plot of MeanNN during Rest. Bland-Altman Plot of MeanNN during Rest. (PDF 136 kb) [file 12911_2019_742_MOESM2_ESM.pdf]

## MeanNN during Stress

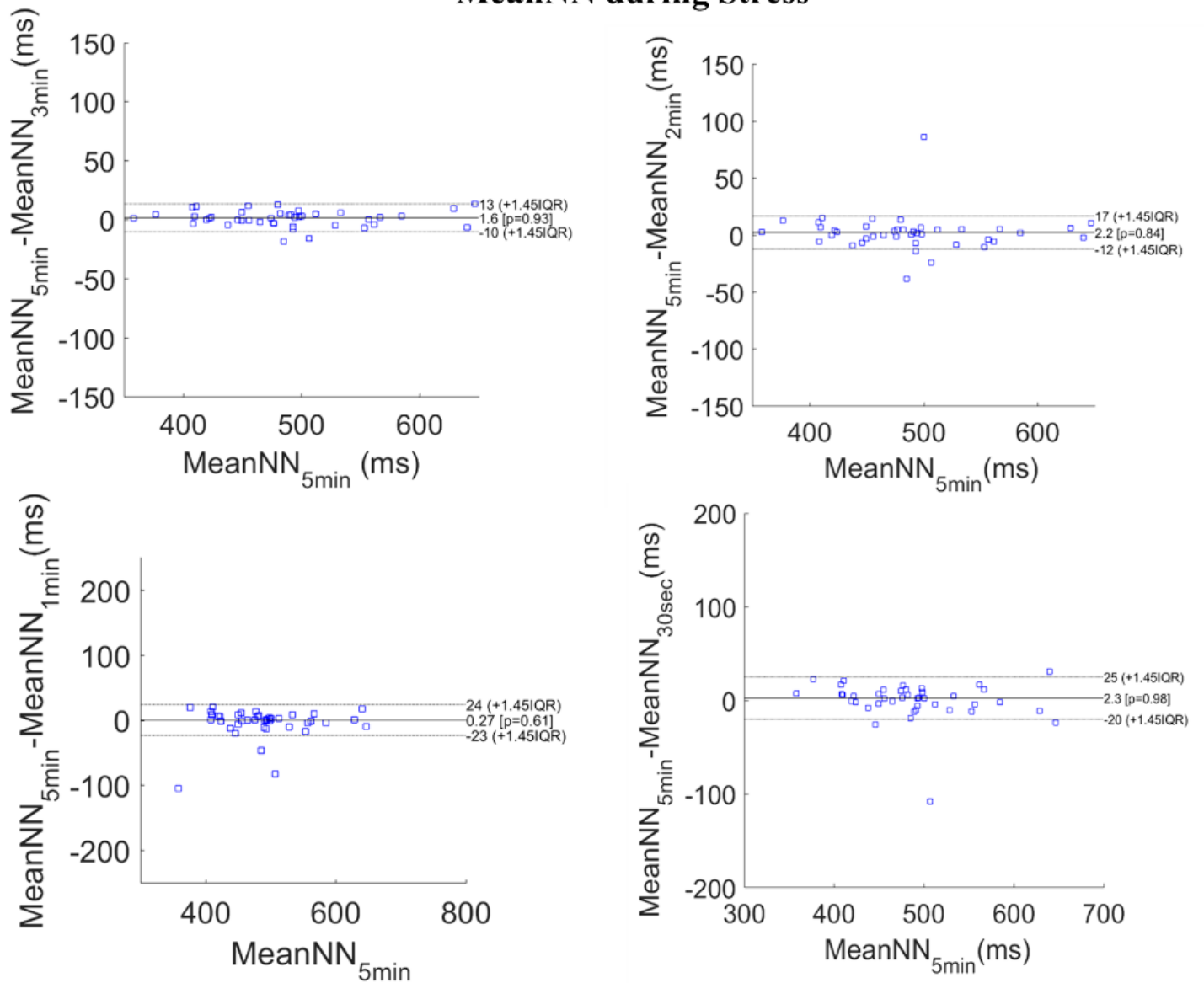

Fig. S2 Bland-Altman Plot of MeanNN during Stress. IQR: Interquartile range.

Supplement: Supplementary file 3 — Figure S2. Bland-Altman Plot of MeanNN during Stress. Bland-Altman Plot of MeanNN during Stress. (PDF 207 kb) [file 12911_2019_742_MOESM3_ESM.pdf]
